# Insights into the roles of bacterial infection and antibiotics in Parkinson’s disease

**DOI:** 10.3389/fcimb.2022.939085

**Published:** 2022-07-28

**Authors:** Shuo Sheng, Shuo Zhao, Feng Zhang

**Affiliations:** ^1^ Key Laboratory of Basic Pharmacology of the Ministry of Education, Zunyi Medical University, Zunyi, China; ^2^ Joint International Research Laboratory of Ethnomedicine of the Ministry of Education, Zunyi Medical University, Zunyi, China; ^3^ Key Laboratory of Basic Pharmacology of Guizhou Province, Zunyi Medical University, Zunyi, China; ^4^ Electron Microscopy Room of School of Basic Medicine, Zunyi Medical University, Zunyi, China; ^5^ Laboratory Animal Center, Zunyi Medical University, Zunyi, China

**Keywords:** Parkinson’s disease, bacterial infection, neuroinflammation, antibiotics, anti-PD

## Abstract

Parkinson’s disease (PD) is one of the most common neurodegenerative disorders, which is accompanied with the classical motor symptoms and a range of non-motor symptoms. Bacterial infection affects the neuroinflammation associated with the pathology of PD and various antibiotics have also been confirmed to play an important role not only in bacterial infection, but also in the PD progression. This mini-review summarized the role of common bacterial infection in PD and introduced several antibiotics that had anti-PD effects.

## Introduction

Parkinson’s disease (PD) is one of the most common neurodegenerative diseases, which seriously affects patients’ health and quality of life. The clinical manifestations of PD include non-motor symptoms and motor symptoms. Motor symptoms are mainly motor retardation and static tremor, while non-motor symptoms include sleep disorder, smell loss, anxiety, depression and cognitive disorder (Homayoun, 2018). Levodopa is currently the first choice of the treatment of PD, but long-term use of levodopa will cause obvious adverse reactions, and thus could not achieve a complete cure effect (Tambasco et al., 2018). The main pathological features of PD are the loss of dopaminergic neurons in the substantia nigra pars compacta (SNpc) and the accumulation of misfolded α-synuclein (Balestrino and Schapira, 2020). Although PD might be associated with several cellular mechanisms, including mitochondrial dysfunction, oxidative stress, neuroinflammation, and defective protein degradation, the pathogenesis of PD is still unclear (Poewe et al., 2017).

It has recently been discovered that bacteria play an important role in the pathogenesis of neurodegenerative diseases (Sampson et al., 2016; Kim et al., 2020). A large number of studies have shown a strong link between bacterial infection and neuroinflammation (Morais et al., 2021; Shen et al., 2022). Neuroinflammation is considered to be one of the causes of PD (Hirsch and Standaert, 2020), and bacterial infections have been confirmed to be closely associated with PD. For example, gastrointestinal infections increased the risk of the disease (Nerius et al., 2020). In this mini review, we summarized the association between multiple bacterial infections and PD, and then discussed the role of antibiotics in the treatment of PD.

## Effects of bacterial infection on PD pathogenesis

**Figure 1:**
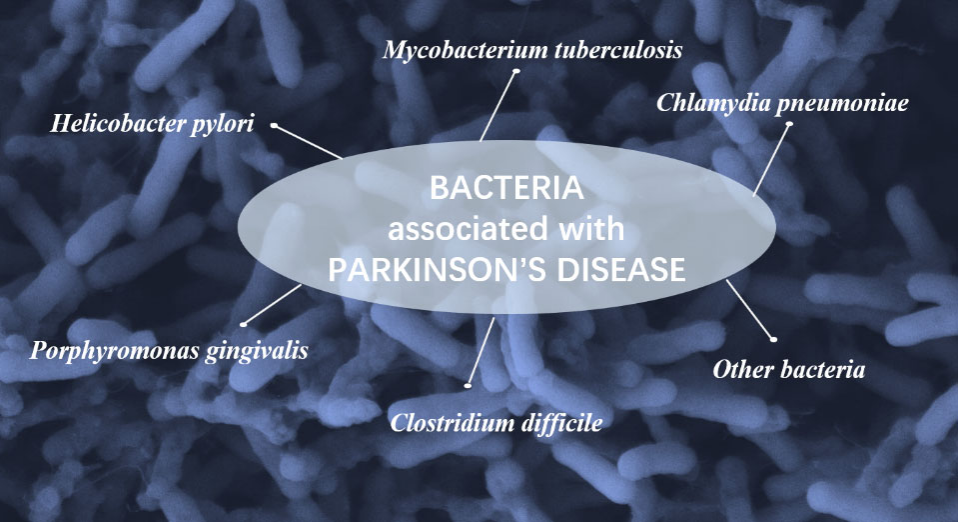
Main bacteria associated with PD.

Bacteria could cause a variety of infections, most commonly in the lung, skin and gastrointestinal tract, etc. (Alby and Nachamkin, 2016; Cookson, 2017; Deusenbery et al., 2021). Several bacterial infections are closely related to the onset of PD (**Figure 1**).

### Helicobacter pylori

*Helicobacter pylori (H. pylori)* is a major cause of gastritis, ulcers, gastric adenocarcinoma and MALT type lymphoma, accompanied by a variety of gastrointestinal symptoms (McGee et al., 2018). Current studies indicate that *H. pylori* is associated with neurodegenerative diseases (Miklossy, 2011). *H. pylori* infection is very common in patients with PD (Blaecher et al., 2013). It has long been found that patients with *H. pylori* ulcers are more likely to develop PD than healthy people of the same age (Actis, 2019). Neuroinflammation, autoimmunity and apoptosis induced by *H. pylori* infection might be related to the pathogenesis of PD. In addition, in the treatment of PD with levodopa, the elimination of *H. pylori* infection with antimicrobial could increase the absorption of levodopa in the intestinal tract (Nyholm and Hellström, 2021). However, another evidence demonstrates that eradication therapy for *H. pylori* does not reduce the risk of PD, even though *H. pylori* is a risk factor for PD (Huang et al., 2018). The mechanism of association between *H. pylori* infection and PD is still unclear. The main reason is that there are too many possibilities for *H. pylori* to cause PD, including the toxic factors, the inflammatory reaction and its influence on intestinal flora, etc. (Dobbs et al., 2016; Noto and Peek, 2017). Thus, the epidemiological investigations are needed for further study.

### Mycobacterium tuberculosis

*Mycobacterium tuberculosis (M. tuberculosis)* is a highly infectious bacterium, and known to induce human tuberculosis (TB). A statistical meta-analysis of human gene expression in response to *M. tuberculosis* infection identified several enriched pathways, such as the LRRK2 pathway in PD (Wang et al., 2018), which play a critical role in regulating the central nervous system (CNS) immune milieu in PD patient (Cookson, 2017; Kim and Alcalay, 2017). Moreover, *M. tuberculosis* infection could induce neuroinflammation in astrocytes of PD-related brain regions in a LRRK2-dependent manner. Furtherly, the LRRK2 inhibitors are considered as a major drug development in treatment of PD patients by elevating levels of cytosolic mtDNA and chronic cGAS signaling (Arru et al., 2016; Weindel et al., 2020). Likewise, Rifampicin, an antibiotic commonly used to treat infections with *M. tuberculosis*, was discovered to have the ability of neuroprotective effects by reducing microglial activation and improving neuron survival against inflammation, which provides a novel therapeutic strategy of anti-Parkinson (Yulug et al., 2014; Liang et al., 2017). Recently, a therapeutic strategy with repeating bacillus Calmette-Guerin (BCG) vaccination was found to be applicable in disease with inadequate aerobic glycolysis including PD (Faustman, 2020). Therefore, basing on the therapeutic strategies with *M. tuberculosis* might provide a new mentality in PD treatment.

### Porphyromonas gingivalis

*Porphyromonas gingivalis (P. gingivalis)* is a keystone pathogen for periodontitis (Hajishengallis et al., 2012). Patients with periodontal inflammatory disease (PID) are more likely to develop PD (Chen et al., 2017). It has been demonstrated that inflammation is associated with neurodegenerative diseases, and PD patients have higher levels of inflammatory cytokines in the brain compared with people don’t have PD (Adams et al., 2019). Gingipains are critical proteases encoded by *P. gingivalis* that could interfere or evade the host complement system. Gingipain proteases produce effects on fibrinogen that increases the risk of periodontal bleeding in patients with periodontitis (Haditsch et al., 2020; Kadowaki, 2021). Studies also found that the enzymes interfered coagulation through interacting with fibrinogen, prothrombin (Imamura et al., 2001) and the stimulation of the kallikrein/kinin pathway (Hočevar et al., 2018; Mo et al., 2020). Furthermore, amyloid fibrin (ogen) protein structure was observed in platelet poor plasma clots, and samples from PD patients contain much more amyloid-specific signal compared with the control donors (Adams et al., 2019). Thus, *P. gingivalis* might affect the development of PD by inducing inflammation and blood changes according to the latest research progress.

### The other bacteria

There are many other bacteria verified to be associated with PD. *Clostridium difficile (C. difficile)* is one of the main pathogens causing diarrhea and pseudomembranous colitis which could colonize when the host has intestinal flora (Leffler and Lamont, 2015; Smits et al., 2016). The individuals with *C. difficile* infection (CDI) history were at higher risk of PD during the first 2 years since CDI diagnosis over a Swedish population-based cohort study (Kang et al., 2020), but there was no obviously increased PD risk in long-term follow-up. *Chlamydia pneumoniae (C. pneumoniae)* has been recognized as an important common respiratory pathogen causing otolaryngeal diseases, including pharyngitis, otitis media, tonsillitis and sinusitis (Roulis et al., 2013). *C. pneumoniae* might have close relationship with neurodegenerative, including Alzheimer’s disease (AD) due to its role in protein deposition and apoptosis in CNS (De Chiara et al., 2012). Besides, an epidemiological study demonstrated that PD risk was increased in healthy individuals who have the familiar pathogens, such as *B. burgdorferi* and *C. pneumoniae* (Patrick et al., 2019).

## Antibiotics and PD

Antibiotics are various kinds of chemical compounds that kill directly or inhibit the microorganisms. Antibiotics are used widely in treating bacterial infection diseases and have decreased the mortality rates. Until now, more and more ancillary properties are found in antibiotics, such as anti-inflammatory effects (Moon et al., 2012; Rashed et al., 2022), inducing gastrointestinal motility (Lam & Ng, 2011), and neuroprotective properties against neurodegenerative and neuroinflammatory disorders (Sultan et al., 2013; Ruzza et al., 2014). Thus, antibiotics function as neuroprotective drugs may not only through treating bacterial infections, but also some other approaches. Here, several antibiotics were demonstrated to be the potential alternatives to PD drugs (**Table 1**).

**Table 1:** The protective mechanisms of antibiotics on PD.

Antibiotics	Mechanisms of action	References
Rifampicin	•Increase dopaminergic cell survival	(Bi et al., 2013)
	•Decrease the expression of inflammatory mediators induced by LPS	(Molloy et al., 2013)
Tetracyclines	•Decrease the pro-inflammatory molecules production	(Morris et al., 2018)
	•Decrease matrix metalloproteinase activity	(Cathcart and Cao, 2015)
	•Reduce ROS production	(Romero-Miguel et al., 2021)
β-lactam	•Reduce oxidative damage	(Bisht et al., 2014)
	•Attenuate the degeneration of dopaminergic neurons	(Ho et al., 2014)
	•Inhibit neuroinflammation	(Kaur and Prakash, 2017)

### Rifampicin

Rifampicin, a wide-spectrum antibiotic, is a semisynthetic derivative of rifamycin with the common structure of an naphthohydroquinone chromophore spanned by an aliphatic ansa chain that mainly transporting the drug to across the blood-brain barrier (BBB) into brain parenchyma. Rifampicin has been confirmed to have the apparent protection in neurodegenerative diseases by different multiple mechanisms, including with anti-apoptotic, anti-inflamatory and anti-oxidant properties (Yulug et al., 2014). In addition, rifampicin could increase the number of surviving dopaminergic neurons at different concentrations (Bi et al., 2013). Also, rifampicin pretreatment led to a dose-dependent increase in cell viability of dopaminergic neurons. Meanwhile, rifampicin decreased LP-induced expression of pro-inflammatory mediators (Molloy et al., 2013). Thus, as a macrocyclic antibiotic for the treatment of *M. tuberculosis* and other mycobacterial infections, rifampicin is supported to be a novel anti-inflammatory drug for PD, but the molecular and cellular mechanisms still need further investigations.

### Tetracyclines

Tetracyclines and its derivatives are broad-spectrum antibiotics with inhibitory effect on most gram positive and negative bacteria and the ability of bactericidal in high concentration. In addition to the antibiotic functions, tetracyclines are reported to generate protection against neurodegenerative and neuropsychiatric diseases (Stoilova et al., 2013; Ruzza et al., 2014; Bortolanza et al., 2018) by reducing pro-inflammatory molecule production (Sultan et al., 2013; Morris et al., 2018), inhibiting matrix metalloproteinase activity and mitochondrial dysfunction (Cathcart and Cao, 2015). Furthermore, tetracycline derivatives, including doxycycline (DOX) and minocycline (MIN), are considered as an alternative therapy strategy in neurodegenerative disorders (Reglodi et al., 2015; Socias et al., 2018). Current evidence indicated that MIN mainly inhibited microglial activation, neuronal apoptosis and reactive oxygen species (ROS) production (Romero-Miguel et al., 2021). Moreover, DOX was confirmed to downregulate the expression of matrix metalloproteinases (MMPs) (Cho et al., 2011). Meanwhile, DOX could suppress the activation of microglia (Santa-Cecília et al., 2016). Therefore, there is rapidly growing evidence showing that tetracycline has the potential therapeutic benefit for PD, but clinical studies are needed to confirm its neuroprotective effect.

### β-lactam

Ceftriaxone (CEF) is a β-lactam antibiotic which is most frequently used in local/systemic infection and hospital acquired infections. Recently, CEF have been highlighted the therapeutic efficacy against neurodegenerative diseases. For instance, CEF could ameliorate abnormal uncontrolled movements (Chotibut et al., 2017) in animal models of PD. Moreover, CEF attenuated oxidative damage (Bisht et al., 2014). Also, CEF was found to prevent the degeneration of dopaminergic neurons (Ho et al., 2014) and inhibit neuroinflammation (Kaur & Prakash, 2017). Thus, CEF is currently becoming a research hotspot with its multiple activities to relieve symptoms of PD. At present, more and more studies are re-interested with antibiotics due to its surprising ancillary properties in anti-inflammatory effects. With the affection of variety mental and neurological diseases in human people, drug reuse is considered as a promising new drug discovery strategy basing on the limitation of target-based drugs approaches (Lee and Kim, 2016; Corsello et al., 2017; Gooch et al., 2017).

## Conclusion

Parkinson’s disease is the second most common neurodegenerative disease in the world and levodopa remains the main option for the treatment of PD. In this mini review, the relationship between common bacterial (*H. pylori, M. tuberculosis, P. gingivalis, C. difficile and C. pneumoniae)* infection with PD and their possible action mechanisms, such as neuroinflammation factors, LRRK2 pathway and toxic protein aggregations, were revealed. Meanwhile, the use of antibiotics in treatment of PD is worth exploration, which could provide new strategies in PD treatment. It is worth noting that levodopa is usually administered orally or enterally and the intestinal microbiota could also affect its therapeutic efficacy. Combined use of levodopa with antibiotics to regulate bacterial infection in PD patients might open a new direction to improve the therapeutic effect of levodopa. Furthermore, the underlying mechanisms of these antibiotics’ action still warrant further illumination.

## Author contributions

SS conceived of the topic. SS and SZ wrote the manuscript. FZ helped editing of the manuscript. All authors contributed to this review and approved the submitted version.

## Funding

The work was supported by special grant of academic new seedling cultivation and innovation exploration from Guizhou Science and Technology Department (Qian Ke He Ping Tai Ren Cai [2018]5772-036 and Qian Ke Ping Tai Ren Cai [2020]-012), Science and Technology Foundation of Guizhou Province (No. ZK[2021]-014), Science and Technology Project of Zunyi city (Zun Shi Ke He HZ Zi [2021] No. 286), Natural Science Foundation of Guizhou Province (Qian Ke He Ji Chu – ZK [2022] No. 604), and Science and Technology Project of Zunyi city (Zun Shi Ke He HZ Zi [2022] No. 372).

## Conflict of Interest

The authors declare that the research was conducted in the absence of any commercial or financial relationships that could be construed as a potential conflict of interest.

## Publisher’s note

All claims expressed in this article are solely those of the authors and do not necessarily represent those of their affiliated organizations, or those of the publisher, the editors and the reviewers. Any product that may be evaluated in this article, or claim that may be made by its manufacturer, is not guaranteed or endorsed by the publisher.
